# Two New Species of the Taxonomically Ambiguous *Mongolodiaptomus birulai* Group from Southwestern China and Southeast Asia (Crustacea, Copepoda, Calanoida, Diaptomidae)

**DOI:** 10.3390/biology14121766

**Published:** 2025-12-10

**Authors:** Rachada Chaicharoen, Laorsri Sanoamuang

**Affiliations:** 1 Department of Science and Mathematics, Faculty of Science and Technology, Rajamangala University of Technology Tawan-ok, Si Racha, Chonburi 20110, Thailand; noonrachada@gmail.com; 2 Laboratory of Biodiversity and Environmental Management, International College, Khon Kaen University, Khon Kaen 40002, Thailand; 3 Applied Taxonomic Research Center, Faculty of Science, Khon Kaen University, Khon Kaen 40002, Thailand

**Keywords:** biodiversity, Cambodia, *Mongolodiaptomus formosanus*, *Mongolodiaptomus longiserratus*, *Mongolodiaptomus parabirulai*, Vietnam

## Abstract

Currently, the synonymy of the two morphologically analogous copepod species, *Mongolodiaptomus birulai* (Rylov, 1922) and *M. formosanus* Kiefer, 1937, remains doubtful. While examining the detailed morphology of diaptomid copepods from China, Cambodia, and Vietnam, we came across two undescribed species whose morphology is closely similar to that of *M. birulai* from Northeast China and *M. formosanus* from Taiwan. In this paper, descriptions of *M. parabirulai* sp. nov. from Yunnan, Southwestern China, and *M. longiserratus* sp. nov. from Cambodia and Vietnam are presented. The first new species, *M. parabirulai* sp. nov., can be distinguished from its congeners in the male P5 by it having the distal outer portion of the second right exopod produce a wing-shaped expansion and its strongly robust left and right basis. The second new species, *M. longiserratus* sp. nov., can be differentiated by the following characteristics in the male: the spiniform process on segment 20 of the right antennule is longer than that of segment 21 and has a serrate outer margin, and the right P5 basis has two (longitudinal and semicircular) hyaline lamellae. Furthermore, the status of *M. birulai* and *M. formosanus* is reviewed and recommended.

## Simple Summary

The problem of whether two copepod species, *Mongolodiptomus birulai* and *M. formosanus*, are the same species or not is still unclear. During our investigation of the detailed morphology of diaptomid copepods from China, Cambodia, and Vietnam, we discovered two unidentified species that closely resemble the morphology of *M. birulai* from Northeast China and *M. formosanus* from Taiwan. In this paper, descriptions of *M. parabirulai* sp. nov. from Yunnan, Southwestern China, and *M. longiserratus* sp. nov. from Cambodia and Vietnam are presented. The first new species, *M. parabirulai* sp. nov., can be distinguished from *M. birulai* and the other congeners in male leg 5 by its thick and strong left and right basis, as well as the distal outer end of the second right exopod, which is expanded like a wing. The second new species, *M. longiserratus* sp. nov., can be differentiated by the following characters in the male: the spiniform process on segment 20 of the right antennule is longer than that on segment 21 and has a serrate outer margin, and the right leg 5 basis has two (longitudinal and semicircular) hyaline lamellae. In addition, the status of *M. birulai* and *M. formosanus* is discussed and suggested.

## 1. Introduction

Copepods represent one of the most diversified groups of micro-crustaceans in aquatic environments. Approximately 13,000 species have been identified globally, with the highest diversity of copepods occurring in marine environments; nevertheless, at least 2814 species occur in freshwater ecosystems [1]. Members of the family Diaptomidae Baird, 1850, which is part of the Calanoida order, are small-particle feeders and predominantly inhabit inland waters of Asia, Africa, Europe, North America, and South America. Only two species have been recorded in Australia, and there are no records of diaptomid copepods in New Zealand and New Caledonia. They constitute a significant element of most planktonic, benthic, and groundwater communities. The Diaptomidae family is productive in various freshwater habitats, with 63 genera and over 440 species [2]. At least 92 species have been recorded from the Oriental zoogeographic region, which generally refers to countries in East, South, and Southeast Asia today [1].

In the lower Mekong River basin countries of Southeast Asia, Thailand is the most species-rich of diaptomid copepods, with 45 species identified [3,4,5,6,7], while Vietnam and Cambodia contain 33 and 24 species [8,9,10], respectively. Among these, *Mongolodiaptomus* Kiefer, 1937, and *Tropodiaptomus* Kiefer, 1932, are the most diverse genera, comprising 11 [6,7] and 10 recognized species [11,12,13] in Thailand, respectively. At least eight and five species of *Mongolodiaptomus* have been found so far in Vietnam [8,9] and Cambodia [10], respectively.

The genus *Mongolodiaptomus* was initially created as a subgenus of *Eudiaptomus* by Kiefer [14] and was elevated to generic status by Kiefer [15]. It was established for a group of Asian freshwater diaptomid copepods, with *Mongolodiaptomus formosanus* Kiefer, 1937, as the type species [15]. Currently, *Mongolodiaptomus* comprises 15 recognized species, primarily located in Asia [16], with the lower Mekong River Basin as the focal point [6,17]. Ranga Reddy et al. [18] revised the ornamentation on the right second exopod of the male fifth legs as a significant characteristic for distinguishing diaptomid copepods, particularly within the closely related genera *Neodiaptomus* Kiefer, 1932; *Allodiaptomus* Kiefer, 1936; and *Mongolodiaptomus*. The classification system employed to differentiate the genus *Mongolodiaptomus* from similar genera is outlined in Sanoamuang and Koompoot [6].

However, the male and female morphology of the type species, *M. formosanus* Kiefer, 1937 [15], from Taiwan, closely resembles that of *M. birulai* (Rylov), which was characterized based on specimens collected in Harbin, northeastern China, by Rylov in 1922 [19]. Consequently, multiple researchers from China [20,21], Taiwan [22,23], and Vietnam [8] classified *M. formosanus* as a synonym of *M. birulai*. Despite this classification, researchers such as Chaicharoen and Sanoamuang [10], Walter and Boxshall [16], Ranga Reddy et al. [18], and Lai et al. [24] considered them to be distinct species.

While examining the detailed morphology of diaptomid copepods from Cambodia and Vietnam, we came across an undescribed species whose morphology is closely similar to that of *M. birulai* and *M. formosanus.* In this paper, a description of *M. longiserratus* sp. nov., also referred to as *M. formosanus* in Chaicharoen and Sanoamuang (2022) [10], is presented. In addition, another taxon, which was previously identified as *M. birulai*, is considered a new species, namely, *M. parabirulai* sp. nov., based on specimens collected from Yunnan, China.

## 2. Materials and Methods

Diaptomid copepod samples were collected qualitatively from various freshwater habitats covering two provinces of Cambodia and one province each in Vietnam and China. Samples were taken using a plankton net with a mesh size of 60 μm. The concentrated samples were then preserved in 4% formaldehyde and 70% ethanol immediately after collection. Specimens were dissected and mounted at 40–100× magnification under an Olympus SZ51 stereomicroscope (Olympus, Tokyo, Japan) the countries of the optical and used device manufacturers are Japan and the Philippines). For illustrations, the habitus and all appendages were dissected and drawn at 400× and 1000× magnification with the aid of a drawing tube mounted to an Olympus CH30 compound microscope (Olympus, Tokyo, Japan, the country of the optical and used device manufacturers is Japan). The CorelDRAW^®^ version 12.0 graphic program was employed for the final version of the illustrated figures.

Specimens for scanning electron microscopy (SEM) were subjected to dehydration in a succession of ethanol concentrations (50%, 70%, 80%, 90%, 95%, 100%) for 15 min at each level. A total of 6 specimens were dehydrated in a critical point dryer and subsequently coated with gold using a sputter coater. The SEM images were captured with a scanning electron microscope (FEI Helios NanoLab G3 CX, FEI Company, Hillsboro, OR, USA; the countries of the optical and used device manufacturers are the USA and The Netherlands).

The following abbreviations are used in both the text and the figures: ae, aesthetasc; Enp, endopod; Exp, exopod; Exp-n, exopodal segment n; Enp-n, endopodal segment n; Pdg1–Pdg5, pedigers 1–5; P1–P5, legs 1–5; sp, spine. The nomenclature and descriptive terminology follow Huys and Boxshall [25], including the numbering of caudal setae (I–VII). Type specimens are deposited at the Thailand Natural History Museum (THNHM) and the Applied Taxonomic Research Center at Khon Kaen University, Thailand.

## 3. Results

### 3.1. Taxonomy

Infraclass Neocopepoda Huys and Boxshall, 1991

Order Calanoida Sars, 1903

Family Diaptomidae Baird, 1850

Sub-family Diaptominae Kiefer, 1932

Genus *Mongolodiaptomus* Kiefer, 1937

Type species. *Mongolodiaptomus formosanus* Kiefer, 1937

#### 3.1.1. *Mongolodiaptomus parabirulai* sp. nov. (Figure 1, Figure 2, Figure 3, Figure 4, Figure 5 and Figure 6)

urn:lsid:zoobank.org:pub:D6C9734D-A577-4A4F-9661-65B30073DACE

**Type locality.** A landscape pool (21°52′25.1544″ N, 101°19′22.1952″ E) at Xishuangbanna Tropical Botanical Garden, Chinese Academy of Sciences, Yunnan Province, China.

**Material examined. *Holotype:*** China; one ♂ (adult); a landscape pool at Xishuangbanna Tropical Botanical Garden, Chinese Academy of Sciences, Yunnan Province, accession number: THNHM-lv-21123; dissected, mounted on one slide in glycerol, covered with a coverslip, and sealed with nail polish, collected on 17 April 2010, leg. Shusen Shu.

***Allotype:*** China; one ♀ (adult); location, date, and collectors as for holotype; accession number: THNHM-lv-21124, completely dissected, mounted on one slide in glycerol, covered with a coverslip, and sealed with nail polish.

***Paratypes:*** China; one ♂ (adult) and one ♀ (adult); date and collectors as for holotype; accession number: THNHM-lv-21125, mounted on one slide in glycerol, covered with a coverslip, and sealed with nail polish, and undissected and preserved in 4% formalin.

**Etymology.** The specific name *parabirulai* is a combination of the Greek prefix *para*-, meaning to resemble, and the specific name *birulai*, referring to the fact that the male P5 of the new species resembles *Mongolodiaptomus birulai* (Rylov, 1922).

**Figure 1:**
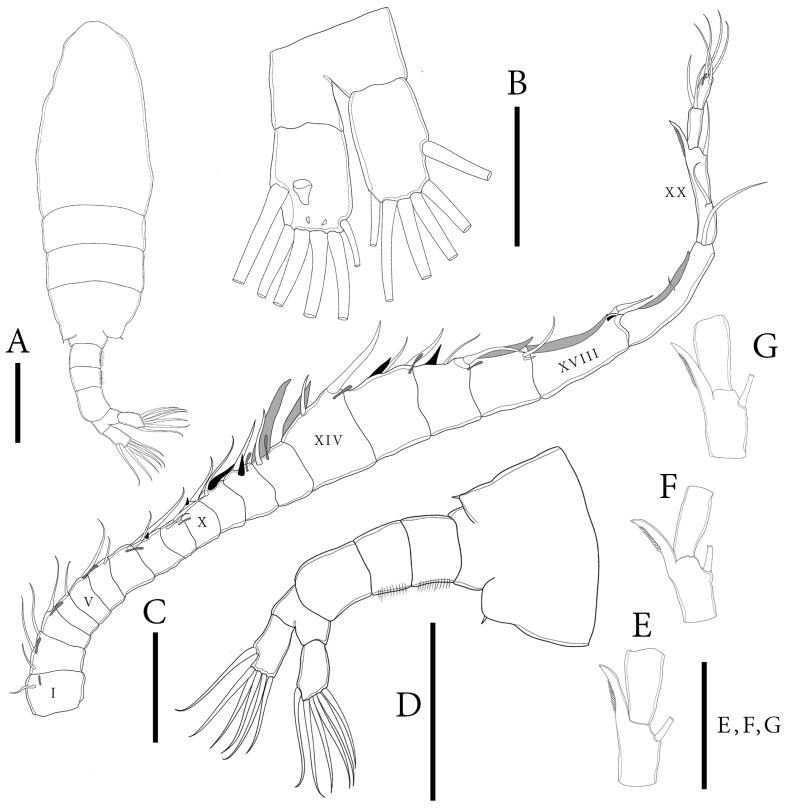
*Mongolodiaptomus parabirulai* sp. nov. male: (**A**) habitus, dorsal view; (**B**) urosome (anal somite) and caudal rami, ventral view; (**C**) right antennule; (**D**) urosome and caudal rami, lateral view; (**E**–**G**) right antennule, distal part of segment 20 showing length variations of spiniform process, and segment 21. Scale bars = 100 µm.

**Description of adult male.** Total body length, measured from anterior margin of rostrum to posterior margin of caudal rami, is 0.70–0.72 mm. (mean = 0.61 mm, *n* = 5); (Figure 1A). Body smaller and more slender than that of females. Prosome ~ 3.0 × as long as urosome (Figure 1D). Pdg4 and Pdg5 completely fused (Figure 1D). Lateral wings of Pdg5 slightly asymmetrical; right postero-lateral wing rounded, larger and shorter than left one; each wing with one thin postero-lateral spine (Figure 1D).

**Urosome** with five somites (Figure 1A,D). Genital somite shorter than its width, lacking spines on posterolateral corners on both sides. Urosomites 2–3 slightly wider than long each. Both urosomites 2–3 with a patch of hairs on ventral side (Figure 1D). Urosomite 4 squarish, has a convex distal end, and longer than urosomites 2–3. Anal somite asymmetrical, with right side longer than left one. Anal somite and caudal rami bent or twisted to right side. Caudal rami appear symmetrical (Figure 1D); each ramus approximately 1.7 times longer than wide, and they have a hairy inner margin. Right ramus armed with three triangular knobs on ventral surface; a large knob located at middle of segment, along with two tiny knobs distally (Figure 1B). Each ramus has six setae, subequal in length and size and plumose: dorsal seta bare and thinner than others.

**Antennules** asymmetrical, extending beyond end of the caudal setae. Left antennule 25-segmented (Figure 3E). Armature formulae as in Table 1. Right antennule geniculated, consisting of 22 segments (Figure 1C). Segment 20 (antepenultimate segment) has a sickle-shaped spiniform process, reaching ~2/3 length of segment 21 (Figure 1C,E–G). Armature formulae as in Table 2.

**Figure 2:**
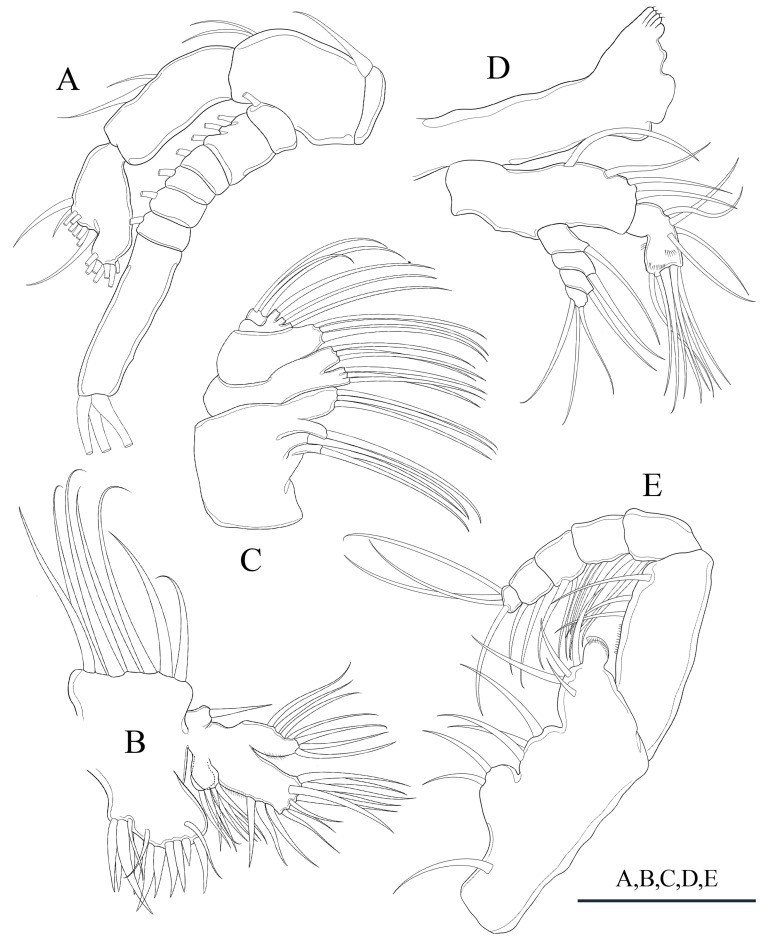
*Mongolodiaptomus parabirulai* sp. nov. male: (**A**) antenna; (**B**) maxillule (**C**) maxilla; (**D**) mandible; (**E**) maxilliped. Scale bar = 100 µm.

**Figure 3:**
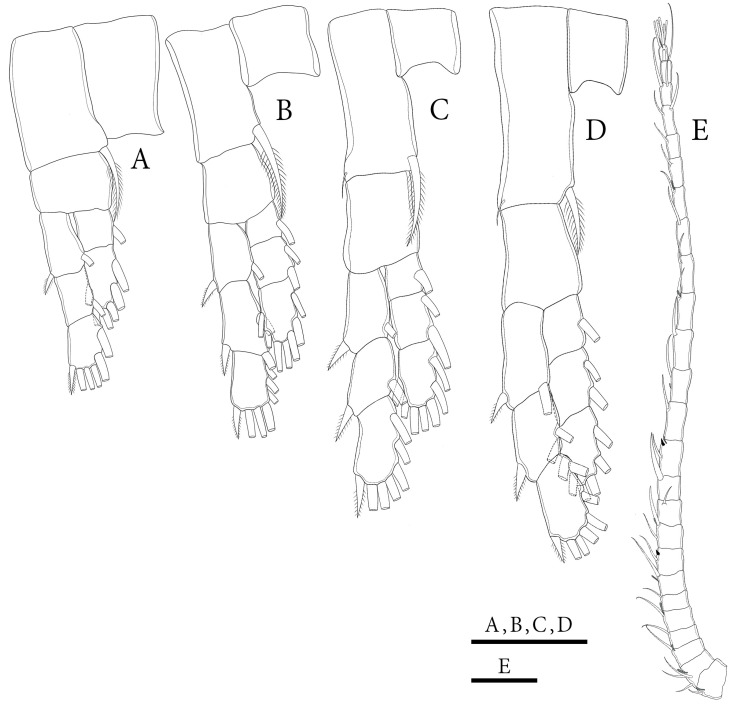
*Mongolodiaptomus parabirulai* sp. nov. male: (**A**) P1; (**B**) P2; (**C**) P3; (**D**) P4; (**E**) left antennule. Scale bars = 100 µm.

**Figure 4:**
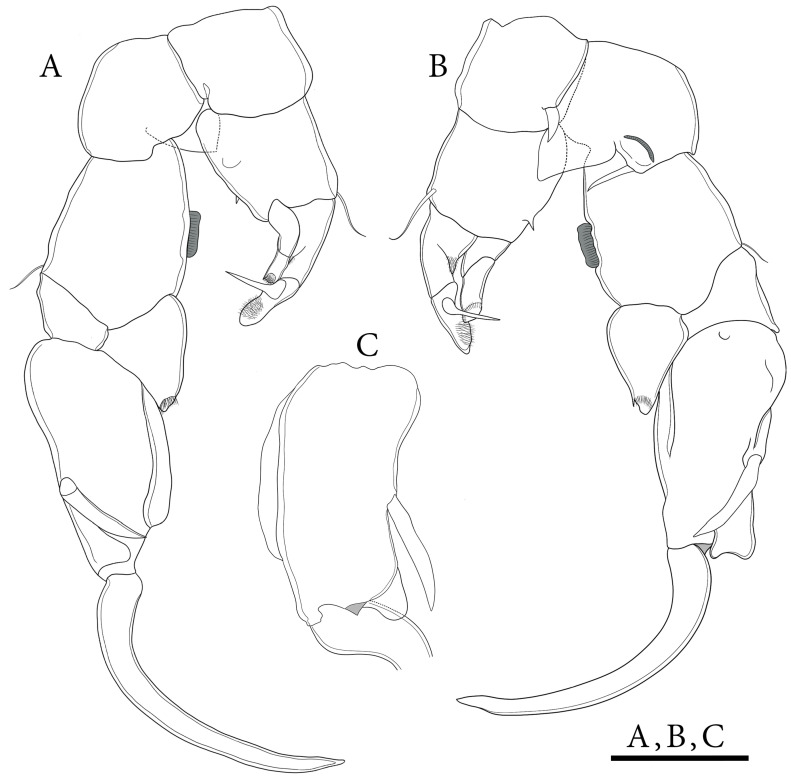
*Mongolodiaptomus parabirulai* sp. nov. male: (**A**) P5, posterior view; (**B**) P5, anterior view; (**C**) right P5, Exp-2, posterior view. Scale bar = 200 µm.

**Figure 5:**
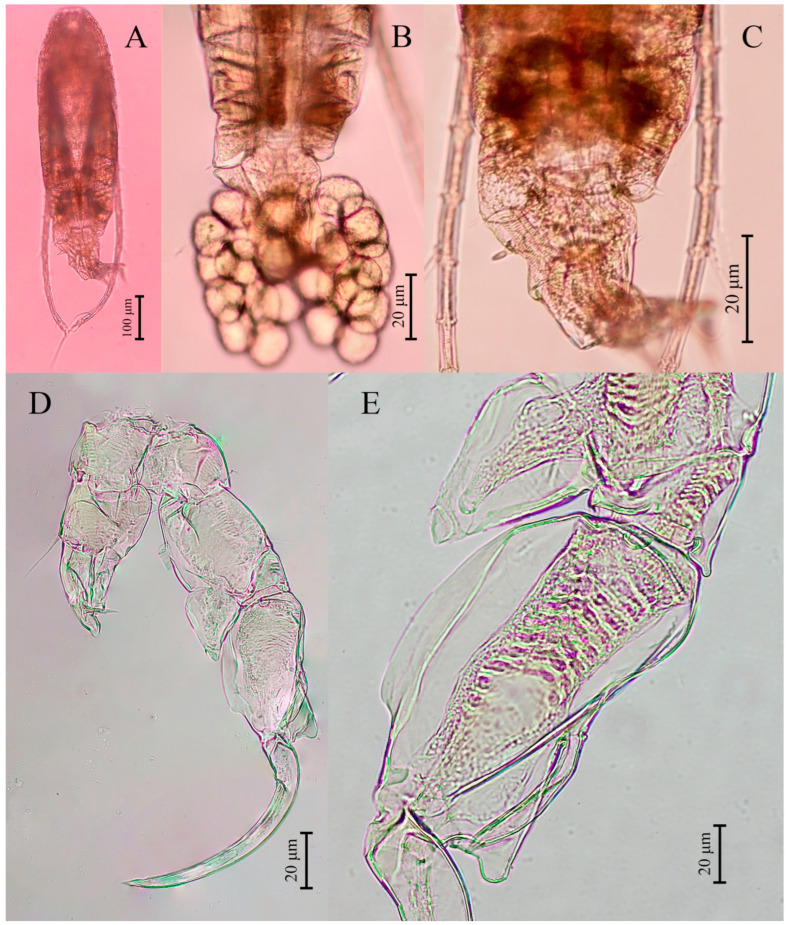
*Mongolodiaptomus parabirulai* sp. nov. female (**A**–**C**) and male (**D**,**E**): (**A**) habitus, dorsal view; (**B**) last pedigerous somites, and urosome, dorsal view; (**C**) last pedigerous somites, urosome and caudal rami, dorsal view; (**D**) P5, posterior view; (**E**) middle part of P5, posterior view.

**Figure 6:**
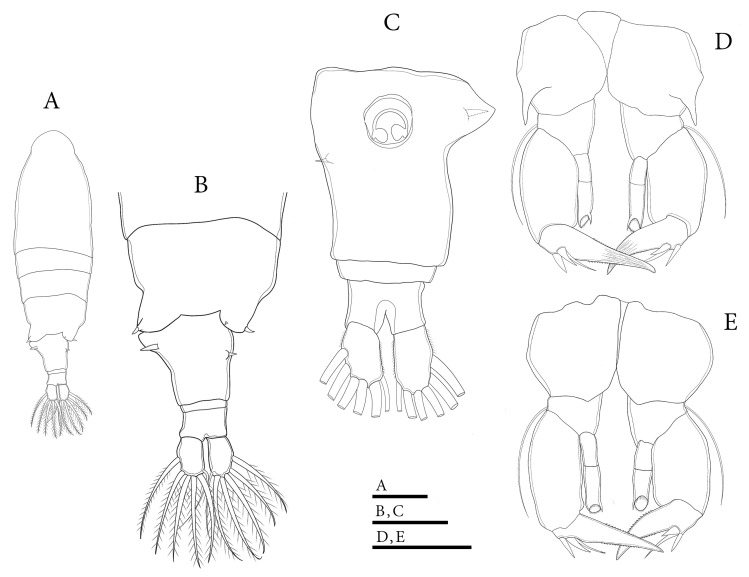
*Mongolodiaptomus parabirulai* sp. nov. female: (**A**) habitus, dorsal view; (**B**) pediger 5, urosome, and caudal rami, dorsal view; (**C**) urosome and caudal rami, ventral view; (**D**) P5, posterior view; (**E**) P5, anterior view. Scale bars = 100 µm.

**Antenna** biramous (Figure 2A). Coxa and basis possess one and two simple setae on inner distal corner, respectively. Enp bifurcated; Enp-1 possesses two setae along inner margin; Enp-2 contains nine setae along inner margin and seven setae apically; all setae lack setal ornamentation. Exp seven-segmented; Exp-1–6 possessing 1, 3, 1, 1, 1, 1 setae along inner margin; Exp-7 exhibiting three setae apically; all setae naked.

**Mandible** (Figure 2D): coxa with ~ five teeth on coxal gnathobase dorsally. Basis with four bare setae; one medially and three distally along inner margin. Enp two-segmented; Enp-1 with four inner setae apically. Enp-2 with nine setae apically and two transverse rows of spinules close to apical end. Exp-1 to Exp-3 each possess one seta on inner border; Exp-4 contains three setae apically; all setae naked.

**Maxillule** (Figure 2B): praecoxal arthrite containing seven robust setae laterally and three delicate submarginal setae. Coxal endite possessing four setae; coxal epipodite containing seven setae; two proximal-most setae smaller than remaining ones. Two basal endites united to a segment that supports them: proximal and distal endite, each possessing four apical setae; basal exopodite contains one short seta. Enp-1 and Enp-2 each possess four setae apically, with proximal segment united to basis. Exp with six naked setae apically.

**Maxilla** (Figure 2C): praecoxa combined with coxa. Proximal endites on praecoxa possess one seta proximally and three setae apically. Distal endites on praecoxa possessing three setae apically. Proximal coxal endites possessing three setae apically. Distal coxal endites possessing three setae apically. Allobasis containing three setae apically. Enp bifurcated into two segments; each bearing three setae.

**Maxilliped** (Figure 2E): four medial lobes on syncoxa, with a setal formula of 1, 2, 3, and 4, respectively; subdistal inner margin expanded into a spherical lobe with a cluster of minute spinules. Basis with a row of tiny spinules proximately and three setae along the inner margin. Enp five-segmented, with 3, 2, 2, 2, and 4 bare setae, respectively.

**P1–P4** (Figure 3A–D): biramous, coxa bearing a pinnate seta at innermost distal corner. Coxa of P3 and P4 with a smaller bare seta on outer distal margin (Figure 3C,D), but the basis lacks setae. Exp longer than Enp; two-segmented Enp and three-segmented Exp on P1, three-segmented Enp and Exp on P2–P4. Armature formula of P1–P4 as in Table 3.

**P5** (Figure 4 and Figure 5D,E): asymmetrical, right leg highly enlarged. Intercoxal sclerite produced into a triangular tongue-like plate at the distal inner corner (Figure 4B). **Right P5**: coxa somewhat squarish with a small, pointed spine, mounted on a small lobe at the distal-medial margin on the posterior surface (Figure 4B and Figure 5D). Basis strongly sturdy and trapezoidal (Figure 4B), ~1.1 × as long as wide, with a narrow, longitudinal hyaline lamella located the medial inner margin (Figure 4B). Sensory seta at the distal corner of the basis are short, barely reaching ~1/4 length of Exp-1 proximally. Exp two-segmented; Exp-1 shorter than wide, outer distal margin unproduced (Figure 4A,B). Exp-2 slightly incurved, with bulging inner margin, outer margin concave, proximal and distal parts of outer margin enlarged, ~2.0 × as long as wide. Distal outer portion of Exp-2 produced into wing-shaped protrusion (Figure 4B,C and Figure 5E). Outer margin of Exp-2 contains principal lateral spine located slightly posterior to mid-length of segment (Figure 4A,B), along with a minor accessory spine located near insertion of end-claw (Figure 4B,C). Principal lateral spine short, ~1/2 length of segment, slightly curved, bent inward towards distal inner margin of Exp-2 segment (Figure 4A,B) or closely pointed against outer margin (Figure 4C). End-claw thick, short, sickle-shaped, ~as long as preceding two segments combined, gently curved, gradually attenuating to blunt extremity. Enp one-segmented, sturdy triangular shaped, reaching ~1/3 length of Exp-2 segment, gradually tapering to distal end, tipped with tiny spinules distally (Figure 4A,B and Figure 5D,E).

**Left P5**: coxa squarish with a small, pointed spine at distal inner corner on posterior surface, (Figure 4B), ~1/2 length of spine on right coxa. Basis sturdy, trapezoidal, proximal portion broader than distal end, with tiny spine on 2/3 length of inner margin (Figure 4A, B); and long, thin posterolateral seta on posterior outer corner, extending ~1/2 length of Exp-1. Exp three-segmented: Exp-1 longer than wide, gradually tapering in posterior end; with inner serrate margin at distal half. Exp-2 conical, smaller than Exp-1; with inner robust seta, longer than Exp-2 (Figure 4A,B) and inner serrate margin. Exp-3 reduced to bare apical process with blunt-tip, ~2/3 length of Exp-2. Enp two-segmented, longer than Exp-1, with spinulated tip (Figure 4A,B).

**Description of adult female.** Total body length, measured from anterior margin of rostrum to posterior margin of caudal rami, 0.65–0.87 mm (mean = 0.80 mm, *n* = 5) (Figure 5A and Figure 6A). Prosome:urosome ratio ~ 2.8:1.0. Prosome similar to that of male. Fourth and fifth pedigerous somites completely fused (Figure 6A,B). Fifth pediger with sub-asymmetrical posterolateral wings (Figure 6A,B); both wings rounded, left wing slightly larger than right wing; left postero-lateral spine larger than left one, right wing with a minute spine on medial inner margin.

**Urosome** (Figure 6B,C) three-segmented, with strongly asymmetrical genital double-somite. Genital double-somite exceeds total length of urosomite 2, anal somite, and caudal rami (Figure 6B,C); strongly asymmetrical, left portion largely expanded than right one, both portions gradually tapering to distal end, left spine located at proximal dilated portion and laterally pointed, right proximal spine laterally pointed. A pair of gonopores and copulatory pores located centrally at ~1/3 length of genital double-somite (Figure 6C). Urosomite 2 smallest, shorter than wide. Anal somite symmetrical, as long as length of caudal rami. Caudal rami parallel, symmetrical; both rami with hairy inner margins (Figure 6C). All principal caudal setae slightly dilated anteriorly; dorsal seta approximately as long as principal setae.

**Antennules** symmetrical; left antennule, antenna, mouthparts, and P1–P4 as in male.

**P5**: asymmetrical (Figure 6D,E). Intercoxal sclerite somewhat wide, triangular in posterior view. Distal outer border of coxa extends posteriorly into spiniform apophysis that reaches distal portion of basis (Figure 6D). Basis contains a slender, bare seta on distolateral margin, extending approximately 4/5 length of Exp-1. Exp three-segmented (Figure 6D,E). Exp-1 sub-rectangular, ~2.0 as long as wide, with a convex outer margin and straight inner margin. Exp-2 symmetrical, triangular shaped, with a row of spinules along both margins. Lateral spine on Exp-2 ~ same length of outer spine on Exp-3. Exp-3 small and united with proximal outer margin of Exp-2, provided with two unequal spiniform setae apically; inner spine possesses finely serrate margins and extending to approximately half the length of Exp-2. Enp one-segmented (Figure 6D,E), elongated rectangle, equivalent in length to Exp-1; containing obliquely truncate and finely spinulose tip.

**Distribution.** *M. parabirulai* sp. nov. has so far been found in the type locality in Yunnan, China. Some specimens that were previously identified as *M. birulai* in the tropical areas of China [20,21] may turn out to be this new species.

#### 3.1.2. *Mongolodiaptomus longiserratus* sp. nov. (Figure 7, Figure 8, Figure 9, Figure 10, Figure 11, Figure 12, Figure 13 and Figure 14)

urn:lsid:zoobank.org:act:3BF49F10-E90A-4F51-A5A9-FA1B06E9B222

*Mongolodiaptomus formosanus*: Chaicharoen and Sanoamuang (2022): 1, 5, 6, 7, 8, 12, 13, 14.

**Type locality.** A temporary pond (13°05′18” N, 106°10′48” E), in Cambodia, Kratie Province, Kratie District.

**Material examined. *Holotype*:** Cambodia; one ♂ (adult); a temporary pond in Kratie Province, Kratie District, accession number: THNHM-lv-21112; dissected, mounted on one slide in glycerol, covered with a coverslip, and sealed with nail polish, collected on 15 February 2007, leg. Rachada Chaicharoen; water temperature 33.4 °C, pH 6.8, and conductivity 93 µS cm^−1^.

***Allotype***: Cambodia; one ♀ (adult); location, date and collectors as for holotype; accession number: THNHM-lv-21113, completely dissected, mounted on one slide in glycerol, covered with a coverslip, and sealed with nail polish.

***Paratypes***: Cambodia; three ♂ (adult) and three ♀ (adult); date and collectors as for holotype; accession number: THNHM-lv-21114, undissected and preserved in 4% formalin.

**Etymology.** The Latin term *serratus* means *toothed like a saw*. Thus, the specific name *longiserratus* refers to the long and serrated spiniform process on the antepenultimate segment of the male grasping antennule. The name is an adjective in the nominative singular, masculine gender.

**Figure 7:**
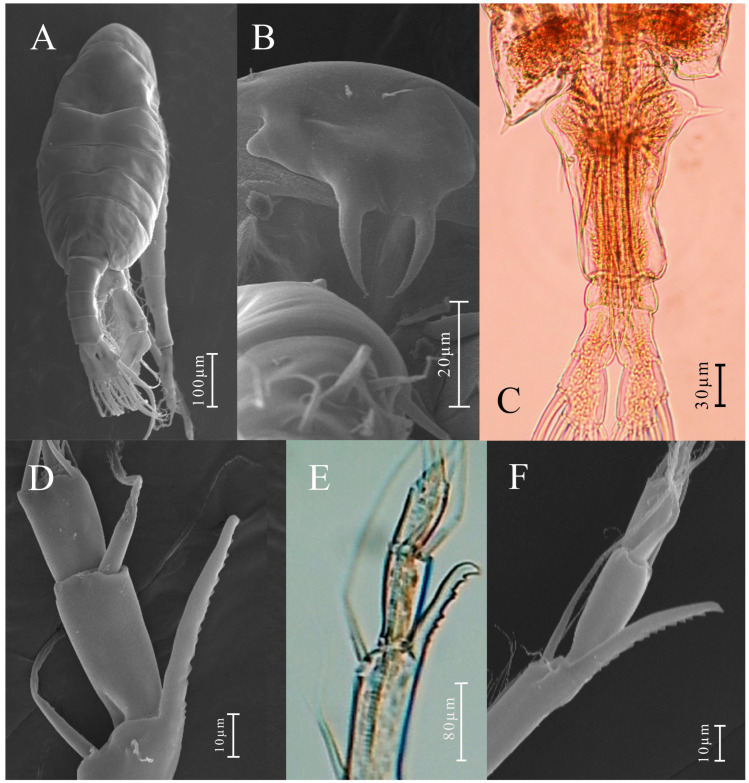
*Mongolodiaptomus longiserratus* sp. nov., SEM and light microscopic photographs of male (**A**,**B**,**D**–**F**) and female (**C**): (**A**) habitus, dorsal view; (**B**) rostrum; (**C**) last pedigerous somites, urosome and caudal rami, dorsal view; (**D**–**F**) spiniform processes on antepenultimate segment of right antennule.

**Figure 8:**
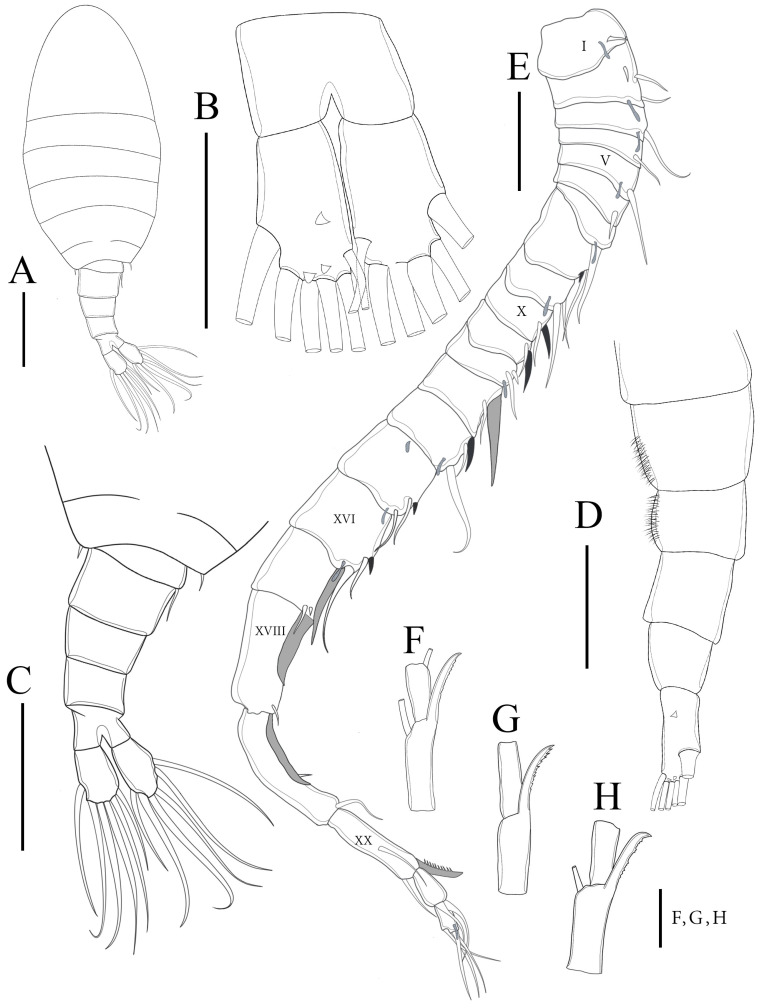
*Mongolodiaptomus longiserratus* sp. nov. male: (**A**) habitus, dorsal view; (**B**) urosome (anal somite) and caudal rami, ventral view; (**C**) pediger 5, urosome, and caudal rami, dorsal view; (**D**) urosome and caudal rami, lateral view; (**E**) right antennule; (**F–H**) right antennule, segments 20–21. Scale bars = 100 µm.

**Description of adult male.** Total body length, measured from anterior margin of rostrum to posterior margin of caudal rami, 0.60–0.62 mm. (mean = 0.61 mm, *n = 5*); (Figure 7A and Figure 8A). Body smaller and more slender than in female. Prosome approximately 2.2 times the length of urosome urosome (Figure 8A). Rostrum well developed, having two spiniform processes (Figure 7B). Cephalosome with transversal groove dorsally at anterior part of somite. Pedigers 4 and 5 incompletely fused (Figure 8A). Lateral wings of Pdg 5 slightly asymmetrical; right postero-lateral wing shorter and smaller than left one; each wing with one thin postero-lateral spine (Figure 8C).

**Urosome** (Figure 8A,C,D) with five somites. Genital somite shorter than wide, having a slender spine at posterolateral corner on right side. Urosomites 2–4 slightly wider than long each. Urosomites 2 and 3 with a patch of hairs on ventral side (Figure 8D). Anal somite asymmetrical and twisted to right side. Caudal rami symmetrical (Figure 8B), with each ramus approximately 0.95 times as long as wide, inner border hairy. Right ramus armed with three small, semicircular, chitinous knobs on ventral surface; one situated on middle of segment and two knobs distally (Figure 8B). Each ramus possesses six setae, about equal in length and size, plumose in nature; dorsal seta lacks of plumosity and thinner than others.

**Antennules**: asymmetrical, extending not beyond end of caudal setae. Left antennule (Figure 10E): 25-segmented. Armature formula as presented in Table 4. Right antennule comprises of 22 segments (Figure 7D–F and Figure 8E–H). Armature formula as presented in Table 5. Segment 20 (antepenultimate segment) possesses an elongated, serrated, sickle-shaped spiniform process extending beyond length of segment 21 (Figure 7D–F and Figure 8F–H).

**Figure 9:**
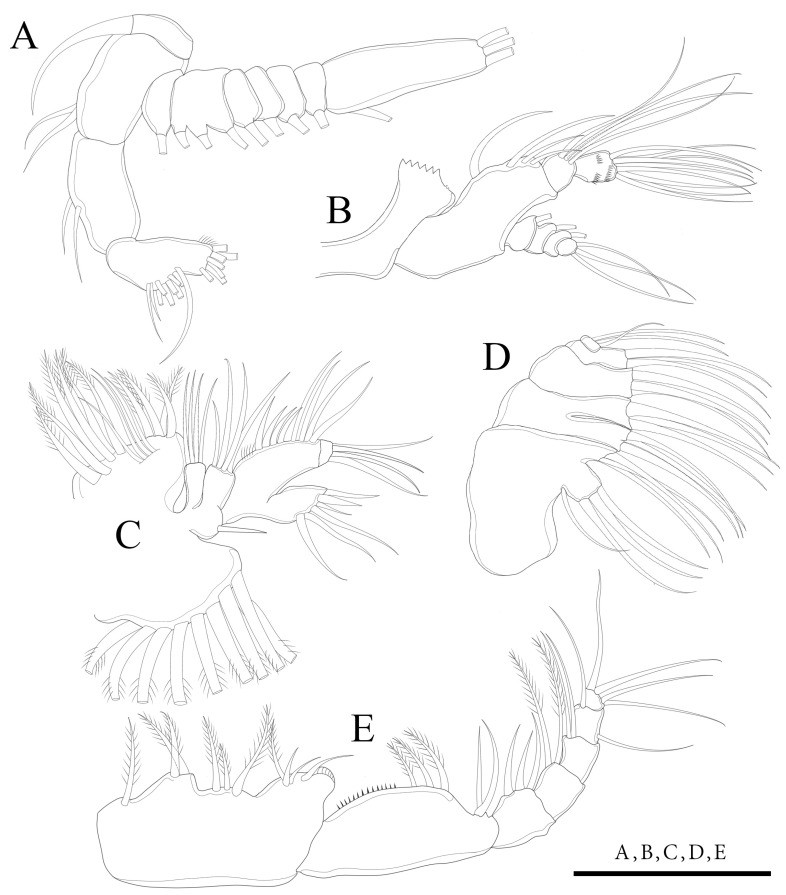
*Mongolodiaptomus longiserratus* sp. nov. male: (**A**) antenna; (**B**) mandible; (**C**) maxillule; (**D**) maxilla; (**E**) maxilliped. Scale bar = 100 µm.

**Antenna** (Figure 9A) biramous. Coxa and basis possess one and two bare setae on inner distal corner, respectively. Enp bifurcated; Enp-1 possesses two setae along inner border; Enp-2 contains nine setae along inner border, seven setae apically; all setae lack of ornamentation. Exp seven-segmented; Exp-1–6 with 1, 3, 1, 1, 1, 1 setae along inner border; Exp-7 has one seta on inner border and three setae apically; all setae naked.

**Mandible** (Figure 9B): coxa with 6 teeth on coxal gnathobase dorsally. Basis with four bare setae; one medially and three distally along inner margin. Enp two-segmented; Enp-1 with four inner setae apically. Enp-2 with nine inner setae apically and three transverse rows of spinules close to apical end. Exp-1–3 each with one seta on inner margin; Exp-4 with three setae apically; all setae bare.

**Maxillule** (Figure 9C): praecoxal arthrite with ten strong setae laterally and four slender submarginal setae. Coxal endite with four setae; coxal epipodite with nine setae; two proximal-most setae smaller than others. Two basal endites fused to segment bearing them: proximal and distal endite, each with four setae apically; basal exopodite with one short seta. Enp-1 and Enp-2 each with four setae apically, proximal segment fused to basis. Exp with six bare setae apically.

**Figure 10:**
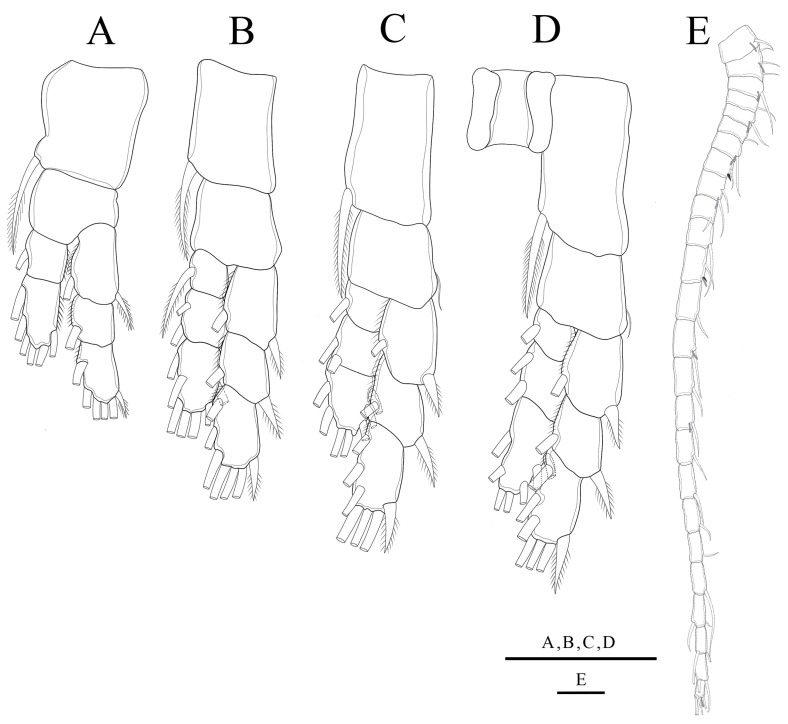
*Mongolodiaptomus longiserratus* sp. nov. male: (**A**) P1; (**B**) P2; (**C**) P3; (**D**) P4; (**E**) left antennule. Scale bars = 100 µm.

**Figure 11:**
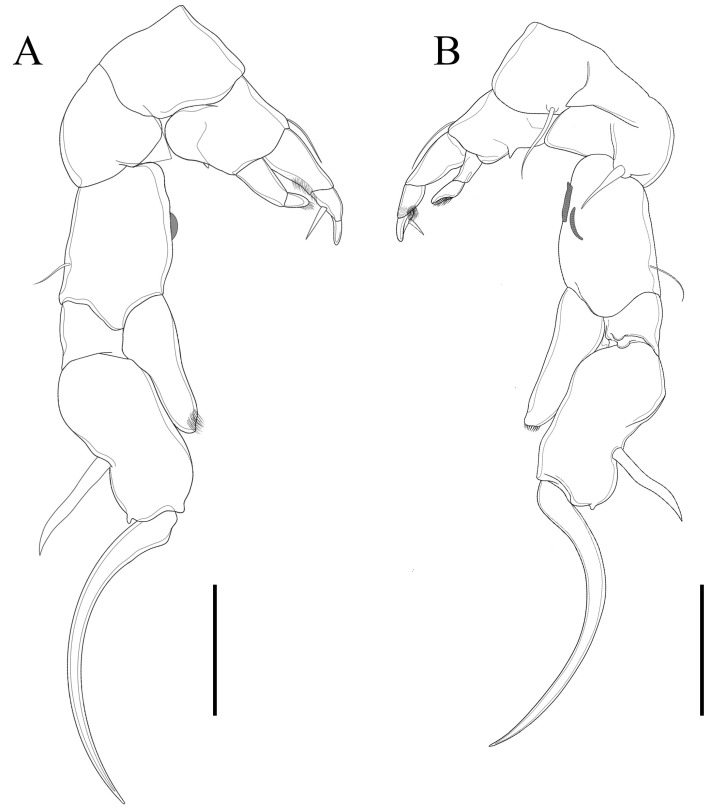
*Mongolodiaptomus longiserratus* sp. nov. male: (**A**) P5, posterior view; (**B**) P5, anterior view. Scale bars = 200 µm.

**Figure 12:**
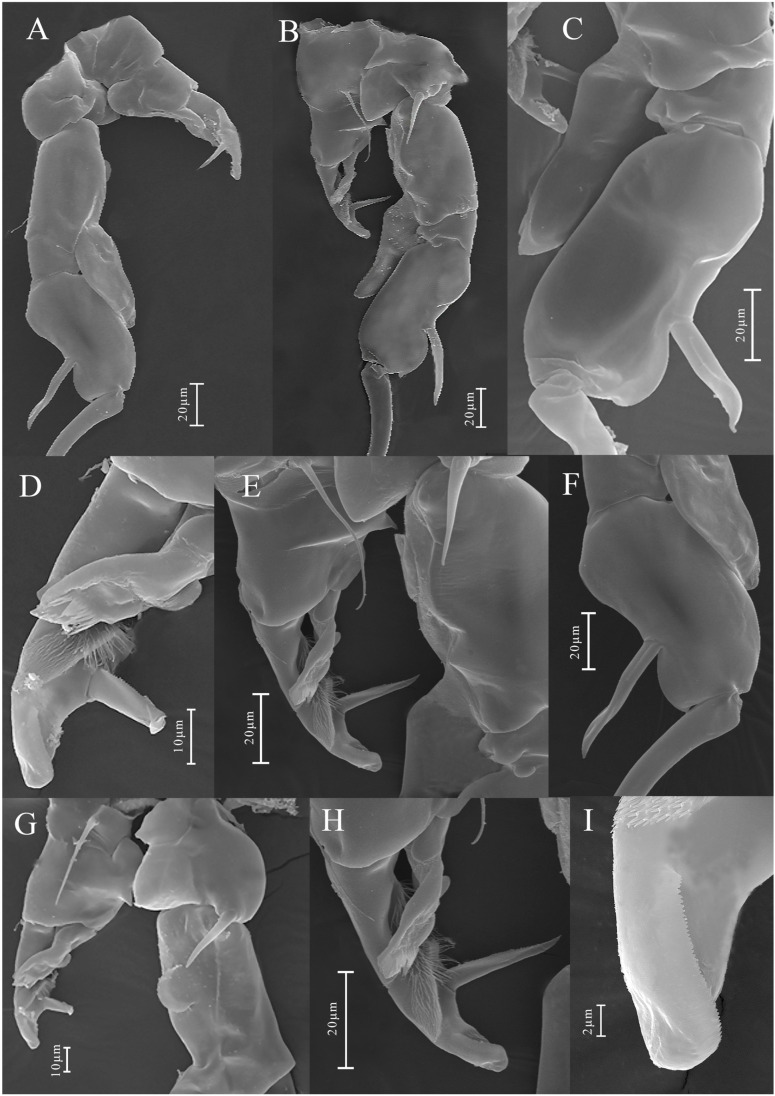
*Mongolodiaptomus longiserratus* sp. nov., SEM photographs of male P5: (**A**) P5, anterior view; (**B**) P5, posterior view; (**C**) right P5 distal part of basis, Exp-1–2, and Enp, posterior view; (**D**) distal part of left P5, posterior view; (**E**) left P5 and proximal part of right P5, posterior view; (**F**) right P5 Exp-1–2, and Enp, anterior view; (**G**) left P5 and proximal part of right P5, posterior view; (**H**,**I**) distal part of left P5, posterior view.

**Figure 13:**
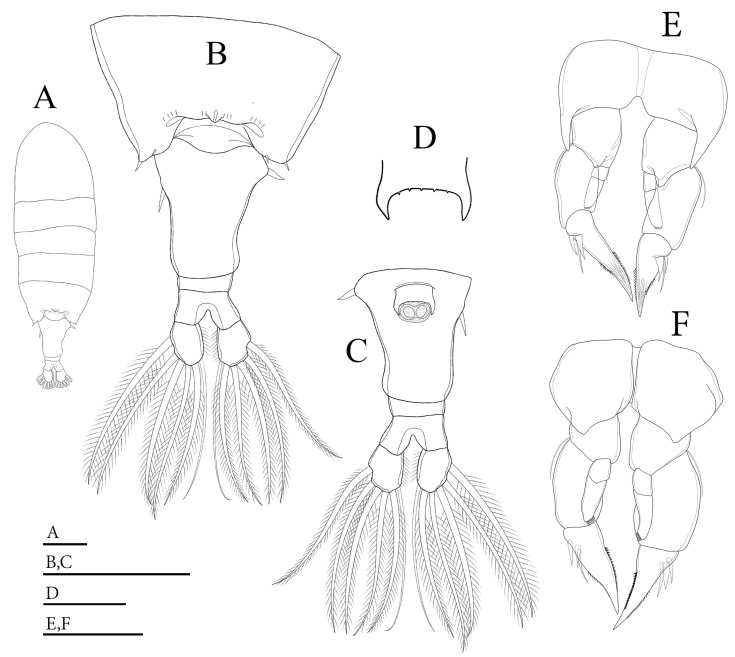
*Mongolodiaptomus longiserratus* sp. nov. female: (**A**) habitus, dorsal view; (**B**) pediger 5, urosome, and caudal rami, dorsal view; (**C**) urosome and caudal rami, ventral view; (**D**) genital operculum; (**E**) P5, posterior view; (**F**) P5, anterior view.

**Figure 14:**
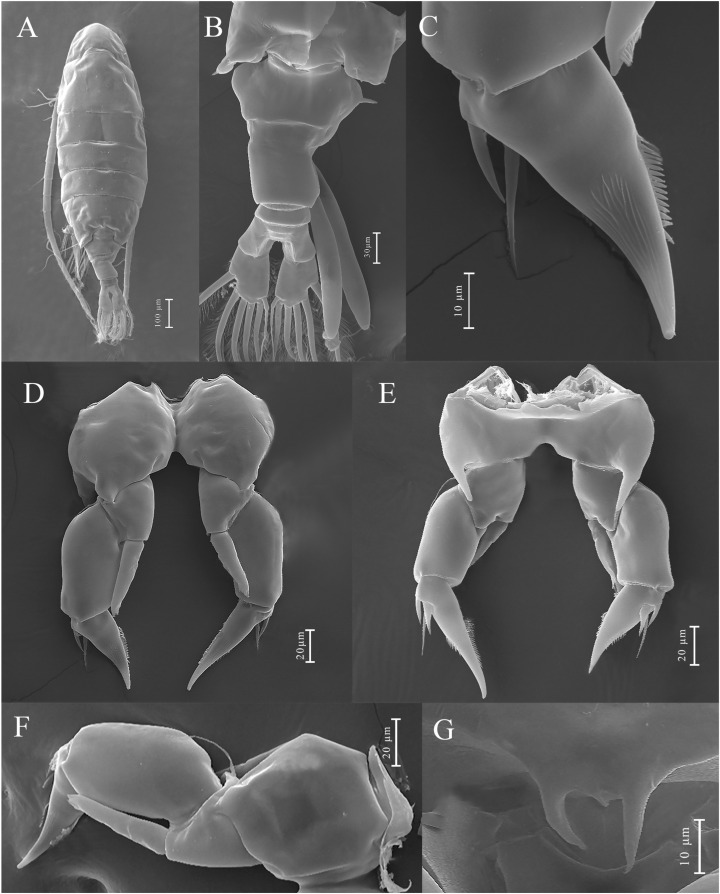
*Mongolodiaptomus longiserratus* sp. nov., SEM photographs of female: (**A**) habitus, dorsal view; (**B**) pediger 5, urosome, and caudal rami, dorsal view; (**C**) P5 Exp-1–2, posterior view; (**D**) P5, posterior view; (**E**) P5, anterior view; distal part of left; (**F**) left P5, posterior view; (**G**) rostrum.

**Maxilla** (Figure 9D): praecoxa united with coxa. Proximal endites on praecoxa possess one seta proximally and three setae apically. Distal endites on praecoxa possess three setae apically. Two coxal endites, each bearing three apical setae. Allobasis possessing three apical setae. Enp bifurcated into two segments; each bearing three setae.

**Maxilliped** (Figure 9E): four medial lobes on syncoxa, with setal formula 1, 2, 3, 4, respectively; subdistal inner border extends into a spherical lobe bearing a patch of minute spinules. Basis with a row of minute spinules proximately, three setae along medial inner border, and two bare setae apically. Enp composed of five segments, bearing 3, 2, 2, 2, and 4 bare setae, respectively.

**P1–P4** (Figure 10A–D): biramous, with coxa bearing a pinnate seta at innermost distal corner. P1 and P2 basis lacking setae; but having a reduced bare seta on outer distal border of P3 and P4. Exp longer than Enp; two-segmented Enp and three-segmented Exp on P1, three-segmented Enp and Exp on P2–P4. Armature formula of P1–P4 as presented in Table 6.

**P5** (Figure 11 and Figure 12): asymmetrical, right leg highly enlarged. Intercoxal sclerite produced into two structures; a smaller triangular spine at proximal-medial corner and a larger tongue-like plate at distal inner corner (Figure 11B,D and Figure 12E,G). **Right P5**: coxa somewhat squarish with a moderately large, pointed spine, mounted on large lobe at distal-medial margin on posterior surface (Figure 11B and Figure 12B). Basis rectangular (Figure 11B and Figure 12A,B,G), ~1.7 × as long as wide; with a narrow, longitudinal hyaline lamella on proximal inner margin (Figure 11B and Figure 12B,E), and a semicircular hyaline lamella at middle inner margin (Figure 11B and Figure 12E,G). Sensory seta at distal corner of basis short, barely reaching ~1/3 length of Exp-1 segment proximally. Exp two-segmented; Exp-1 narrower than wide, having two chitinous knobs at distal inner corner; outer distal border extended into sharp tip (Figure 11A,B and Figure 12A,B). Exp-2 slightly incurved, inner border convex, proximal and distal regions of outer border expanded, and overall length approximately twice the width. Outer border has a principal lateral spine located slightly posterior to midpoint of segment (Figure 11A,B and Figure 12C,F), along with a small accessory spine located near insertion point of end-claw (Figure 11B and Figure 12B). Principal lateral spine appears slender (Figure 12A–C), having a straight or slightly curved contour, extending approximately two-thirds of segment’s length. End-claw sickle-shaped, elongated, and slender, having a serrated inner border and an acute point; approximately 1.5 times the length of Exp-2. Enp one-segmented, slender truncated cone (Figure 11A,B and Figure 12A–C), ~2/3 length of Exp-2 segment gradually tapering to distal end, tipped with tiny spinules distally.

**Left P5**: coxa with thin seta inserted on posterior lobe at distal inner margin (Figure 11B and Figure 12B,G), reaching beyond 2/3 length of basis. Basis trapezoidal, proximal portion broader than distal one, with tiny spine on mid-length of inner margin (Figure 11B and Figure 12A); and long, thin posterolateral seta on posterior outer corner, extending ~2/3 length of Exp-1. Exp three-segmented: Exp-1 longer than wide, gradually tapering in posterior end; with inner strongly serrate margin at distal half (Figure 11A and Figure 12G,H). Exp-2 conical, smaller than Exp-1; with inner robust seta, longer than Exp-2 (Figure 12G,H) and inner serrate margin. Exp-3 reduced to bare apical process with blunt-tip, ~2/3 length of Exp-2, outer margin folded into two layers as in Figure 12I. Enp two-segmented, slightly longer than Exp-1, with spinulated tip (Figure 12G,H).

**Description of adult female.** Total body length, measured from anterior margin of rostrum to posterior margin of caudal rami, 0.95–1.08 mm (mean = 1.016 mm, *n* = 5) (Figure 13A and Figure 14A). Prosome to urosome ratio approximately 3.1:1.0. Prosome resembling that of male. Rostrum fused, asymmetrical, and sharply pointed (Figure 14G). Fourth and fifth pedigerous somites fully fused (Figure 13A,B). Fifth pediger with small dorso-medial ridge and sub-asymmetrical posterolateral wings (Figure 13B and Figure 14A); right wing convex, whereas left wing triangular and longer than right one. Urosome three-segmented, having almost symmetrical genital double-somite (Figure 13B,C and Figure 14B). Genital double-somite exceeding combined length of urosomite 2, anal somite, and caudal rami (Figure 13B,C and Figure 14B); left and right portions equally dilated, both portions gradually tapering to distal end, left spine located at one-third of segment and distally pointed, right proximal spine laterally pointed. A pair of gonopores and copulatory pores located centrally at ~1/3 length of genital double-somite (Figure 13C). Genital field as in Figure 13D. Urosomite 2 asymmetrical, smallest in size, and shorter than wide. Anal somite symmetrical, approximately equal in length to caudal rami (Figure 13B,C and Figure 14B). Caudal rami parallel, symmetrical; both rami with hairy inner margins (Figure 13B,C). All principal caudal setae slightly dilated anteriorly; dorsal seta approximately as long as principal setae.

**Antennules:** symmetrical; left antennule, antenna, mouthparts, and P1–P4 as in male.

**P5**: asymmetrical (Figure 13E,F and Figure 14C–F). Intercoxal sclerite narrow, triangular. Distal outer margin of coxa extended on posterior surface into spiniform apophysis reaching distal part of basis, right apophysis slightly larger than left one (Figure 13E and Figure 14E). Basis with thin, bare seta on distolateral margin, reaching ~1/3 length of Exp-1. Exp three-segmented (Figure 13E,F and Figure 14D,E). Exp-1 sub-rectangular, ~2.03 × as long as wide, with a convex outer margin and straight inner margin. Exp-2 triangular, left side slender and longer than right one, with a row of spinules along both margins, and longitudinal grooves (conveyor canals) on posterior view (Figure 13E). Lateral spine on Exp-2 slightly shorter than outer spine on Exp-3. Exp-3 small and fused with proximal outer margin of Exp-2, armed with two unequal spiniform setae apically; inner spine with finely serrate margins and extending to ~2/3 as long as Exp-2, outer spine larger and shorter than inner spine. Enp two-segmented (Figure 14F), subconical, as long as Exp-1; with obliquely truncate and finely spinulose apex.

**Figure 15:**
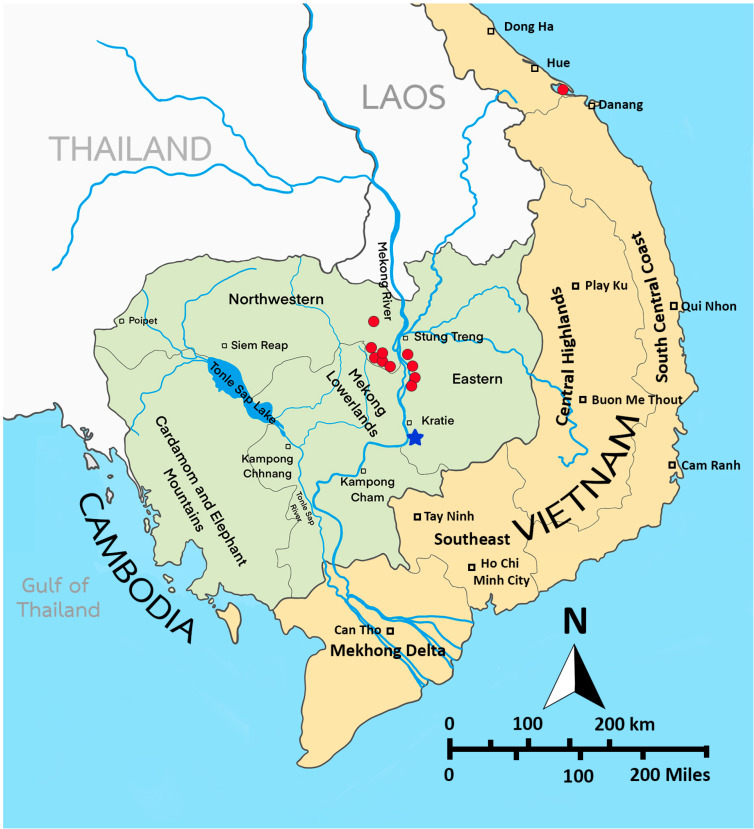
Sampling sites (red spots) of *Mongolodiaptomus longiserratus* sp. nov. in Cambodia and Vietnam. The blue star represents the type locality.

**Distribution.** Cambodia and Vietnam. In Cambodia, it was recorded in 11 of 237 freshwater sampling sites in Kratie and Stung Treng provinces (Figure 15). *M. longiserratus* sp. nov. was found in all three seasons: dry (February), early monsoon (June), and late monsoon (October) [16]. In Vietnam, it has so far been found only in a permanent pond at Lang Co Bay, Phu Loc District, Thua Thien Hue Province, central Vietnam. The new species has been recorded in temporary and permanent ponds, canals, and rivers. Diaptomid species that co-occur with *M. longiserratus* sp. nov. are *Eodiaptomus draconisignivomi* Brehm, 1952; *E. phuvongi* Sanoamuang and Sivongxay, 2004; *Mongolodiaptomus botulifer* (Kiefer, 1974); *M. mekongensis* Sanoamuang and Watiroyram, 2018; *Phyllodiaptomus parachristineae* Sanoamuang and Watiroyram, 2023; *Tropodiaptomus oryzanus* Kiefer, 1937; and *Vietodiaptomus blachei* (Brehm, 1951).

## 4. Discussion

### 4.1. Differential Diagnosis of the Two New Species

The classification of the recognized species of *Mongolodiaptomus* into three separate species groups based on the male features was accomplished by Sanoamuang and Watiroyram [17]. In the next section, there is a presentation of a modified proposal that aims to encompass all of the identified species of *Mongolodiaptomus* into four different groups; for further information, please refer to Sanoamuang & Koompoot (2024) [6]. *Mongolodiaptomus parabirulai* sp. nov. and *M. longiserratus* sp. nov. belong to the *birulai* species group together with *M. birulai*, *M. botulifer*, *M. formosanus,* and *M. malaindosinensis*. The doubtful taxon *M.* cf. *birulai* from the Philippines [24] is also included in this group.

The following characteristics in males are shared by members of the *M. birulai* group: (1) a slender, smooth, or serrate-edged spinous process on the antepenultimate segment of the right antennule; (2) a hyaline lamella on the inner margin of the right P5 basis, but no chitinous prominence; (3) a protruded plate formed from the inner distal margin of the P5 intercoxal sclerite; and (4) ventral chitinous processes on the right caudal ramus.

Based on our specimens of *M. parabirulai* sp. nov. collected from one sampling site in Yunnan Province, China, and *M. longiserratus* sp. nov. from 11 sites in Cambodia and one site in Vietnam (Figure 15), both taxa contain the typical features of the genus *Mongolodiaptomus*, as documented in the revised generic criteria updated by Ranga Reddy et al. [18]. For the male individuals of both species, the right P5 Exp-2 consists of two lateral spines; one principal spine located slightly below the central region of the segment along the outer margin; and the presence of one accessory spine situated distally. Male and female morphological characters of *M. parabirulai* sp. nov. and *M. longiserratus* sp. nov. from this study and the other three congeners from other literature—(1) *M. birulai* from Harbin, China [19,26]; (2) *M.* cf. *birulai* from the Philippines [24]; and (3) *M. formosanus* from Taiwan [15]—are compared in Table 7 and Table 8.

*Mongolodiaptomus parabirulai* sp. nov. and *M. longiserratus* sp. nov. can be distinguished from the other congeners by the following male characteristics (Table 7). The spiniform process on segment 20 of the right antennule in *M. longiserratus* sp. nov. is longer than that of segment 21 and has a serrate outer margin, while that of *M. parabirulai* sp. nov. and the other three taxa (*M. birulai, M.* cf. *birulai,* and *M. formosanus*) is shorter, ~1/2 or 3/4 the length of segment 21, and has a smooth outer margin. The right caudal ramus of *M. longiserratus* sp. nov. has a small, ventral outgrowth on the middle of the segment, plus two small knobs distally, while that of *M. parabirulai* sp. nov. has a large outgrowth, plus two tiny knobs distally. The other three taxa possess an extra-large, large, or small outgrowth in the middle of the segment, but they lack small or tiny knobs distally.

The shape of the male right P5 basis in *M. longiserratus* sp. nov. is a slender rectangle, but that of *M. parabirulai* sp. nov. and *M.* cf. *birulai* is a strongly robust trapezoid, while the other two taxa are sturdy trapezoids. The right P5 basis of *M. parabirulai* sp. nov. and *M.* cf. *birulai* has a particularly lower length-to-width ratio (ranging from 1.1 to 1.2:1.0) than the other three species, which range from 1.5 to 1.7:1.0 (Table 7). The inner margin of the right P5 basis in *M. longiserratus* sp. nov. has two (longitudinal and semicircular) hyaline lamellae, but that of *M. parabirulai* sp. nov. and the other three taxa have only one (longitudinal) hyaline lamella. The shape of the right P5 Enp in *M. longiserratus* sp. nov. is a slender truncated cone and ~ 2/3 the length of the Exp-2, but that of *M. parabirulai* sp. nov. and the other three taxa is a sturdy triangle and ~1/3 the length of the Exp-2.

The shape of the male right P5 Exp-2 in *M. longiserratus* sp. nov. and *M. birulai* lacks a bulging inner margin and has an unproduced distal outer portion, whereas the shape in *M. parabirulai* sp. nov. and *M.* cf. *birulai* from the Philippines includes a bulging inner margin and a distal outer portion that is produced into a distinct wing-shaped expansion. The principal lateral spine of the right P5 Exp-2 in *M. longiserratus* sp. nov. is long and ~2/3 the length of the segment, but that of *M. parabirulai* sp. nov. and the other three taxa is short and ~1/2 the length of the segment. The left P5 Enp in *M. longiserratus* sp. nov. and *M. parabirulai* sp. nov. is two-segmented, while those in the other three taxa are one-segmented.

In the females, *M. longiserratus* sp. nov. and *M. parabirulai* sp. nov. can be differentiated from the other congeners by the following characteristics (Table 8). Both the new species and *M. formosanus* have long antennules, reaching far beyond the end of the caudal setae, while those of *M. birulai* and *M.* cf. *birulai* have shorter antennules, reaching about the end of the caudal setae. There is some overlap of the thoracic and the urosomal lobes in the two new species, *M.* cf. *birulai* and *M. formosanus*, but no such overlap of the lobes in *M. birulai*. The genital double-somite of *M. longiserratus* sp. nov. is almost symmetrical, whereas that of *M. parabirulai* sp. nov. and the other three taxa is strongly asymmetrical. The left and right proximal portions of the genital double-somite of *M. longiserratus* sp. nov. are equally dilated, while only the left portion is largely dilated in *M. parabirulai* sp. nov. and the other three taxa. The left and right spines on the proximal portion of the genital double-somite of *M. longiserratus* sp. nov. are equal in size, and the left spine points distally, while the left spine in *M. parabirulai* sp. nov. and the other three taxa is larger than the right spine, and the left spine points laterally.

The seta on the outer margin of the female P5 basis in *M. longiserratus* sp. nov. and *M. formosanus* is short, reaching ~1/3 or 1/2 of Exp-1, while that of *M. parabirulai* sp. nov. is long, reaching ~3/4 of Exp-1, and that of the other two taxa is very short, reaching ~1/10 of Exp-1. The P5 Enp of *M. longiserratus* sp. nov., *M. parabirulai* sp. nov., and *M.* cf. *birulai* is two-segmented, while that of *M. birulai* and *M. formosanus* is one-segmented.

### 4.2. Taxonomic Status of the M. birulai Species Group

The present study updates a total list of current *Mongolodiaptomus* globally to 16 species. Among the five congeners of the *M. birulai* species group, four are potentially valid, including *M. birulai* (Rylov, 1922), *M. formosanus* Kiefer, 1937, *M. parabirulai* sp. nov., and *M. longiserratus* sp. nov., due to their distinct characteristics (Table 7 and Table 8). *M.* cf. *birulai* from the Philippines [24] morphologically resembles that of *M. parabirulai* sp. nov. by having the following features in the male: (1) the spine on segment 16 of the right antennule is short, (2) the shape of the right P5 basis is a strongly robust trapezoid, (3) the shape of the right P5 Enp is a sturdy triangle, and (4) the shape of the right P5 Exp-2 includes a bulging inner margin and a distal outer portion that is produced into a distinct wing-shaped expansion. The similar female characteristics shared by both species include (1) some overlap between the thoracic and urosomal lobes and (2) a two-segmented P5 Enp. *M.* cf. *birulai*, which may eventually be identified as *M. parabirulai* sp. nov.; however, we cannot conclude the status of this taxon until we examine specimens of *M.* cf. *birulai* from the Philippines.

Kiefer [15] applies four diagnostic characteristics to distinguish *M. formosanus* from *M. birulai*: (1) the extent of convergence of the last thoracic wing from the urosomal lobe in females, (2) the size of ventral outgrowths on the right caudal ramus in males, (3) the morphology and curvature of the antepenultimate hyaline process in the male antennule, and (4) the asymmetry of the left and right fifth legs in females. Thus, we consider the status of *M. formosanus* to be currently valid. Nevertheless, if we are able to determine type specimens from Taiwan, the status of this taxon will be confirmed.

## 5. Conclusions

In this study, we described two morphologically closely resembling copepod species belonging to the *Mongolodiaptomus birulai* species group. Rylov described the oldest species of this group, *M. birulai*, from specimens recorded from Harbin, the northernmost province in Northeastern China, in 1922. Our first new species (*M. parabirulai* sp. nov.) was collected from a landscape pool in Yunnan Province, Southwestern China, and the second new species (*M. longiserratus* sp. nov.) was recorded from eleven sampling sites in Cambodia and another site in Vietnam. We provided a comparison of the most significant morphological characters between the two new species and the other three of their congeners, which were originally found in the three other geographical regions, including the Philippines, Taiwan, and Northeastern China. The differential diagnosis of the two new species is provided, and the status of the five taxa within the *M. birulai* species group is examined. Despite the acknowledgment by various researchers from China, Taiwan, and Vietnam that *M. formosanus* is a synonym of *M. birulai*, we conducted a comparative analysis of morphological traits with other congeners and indicate that they are distinct species. Future analysis of specimens from the type localities of *M. birulai* in Harbin, *M. formosanus* in Taiwan, and *M.* cf. *birulai* in the Philippines will ascertain their validity.

## Figures and Tables

**Table 1:** Armature formula of the left male antennule of *Mongolodiaptomus parabirulai* sp. nov. The numbers of setae (Arabic numerals), aesthetascs (ae), and spines (sp) are given. The Roman numerals refer to segment numbers.

	Segment Number												
	I	II	III	IV	V	VI	VII	VIII	IX	X	XI	XII	XIII
Number of elements	1 + ae	3 + ae	1 + ae	1	1 + ae	1	1 + ae	1 + sp	2 + ae	1	1	1 + ae + sp	1
	XIV	XV	XVI	XVII	XVIII	XIX	XX	XXI	XXII	XXIII	XXIV	XXV	
Number of elements	1 + ae	1	1 + ae	1	1	1 + ae	1	1	2	2	2	5 + ae	

**Table 2:** Armature formula of the right male antennule of *Mongolodiaptomus parabirulai* sp. nov. The numbers of setae (Arabic numerals), aesthetascs (ae), spines (sp), and spiniform processes (spr) are given. The Roman numerals refer to segment numbers.

	Segment Number										
	I	II	III	IV	V	VI	VII	VIII	IX	X	XI
Number of elements	1 + ae	3 + ae	1 + ae	1	1 + ae	1	1 + ae	1 + sp	2 + ae	1 + sp	1 + sp
	XII	XIII	XIV	XV	XVI	XVII	XVIII	XIX	XX	XXI	XXII
Number of elements	1 + ae + sp	1 + ae+ spr	2 + ae + spr	2 + ae+ spr	2 + ae + sp	1 + sp	sp	2	2 + spr	2	4 + ae

**Table 3:** Armature formula for the swimming legs (P1–P4) of *Mongolodiaptomus parabirulai* sp. nov. The quantity of setae (Arabic numerals) and spines (Roman numerals) is represented in the following sequence: outer–inner margin or outer–apical–inner margin.

	Coxa	Basis	Exp			Enp		
			1	2	3	1	2	3
P1	0-1	0-0	I-1	0-1	I-3-2	0-1	I-2-3	- - -
P2	0-1	0-0	I-1	I-1	I-3-3	0-1	0-2	2-2-3
P3	0-1	1-0	I-1	I-1	I-3-3	0-1	0-2	2-2-3
P4	0-1	1-0	I-1	I-1	I-3-3	0-1	0-2	2-2-3

**Table 4:** Armature formula of the left male antennule of *Mongolodiaptomus longiserratus* sp. nov. The quantity of setae (indicated by Arabic numerals), aesthetascs (ae), and spines (sp) is provided. The Roman numerals indicate the segment numbers.

	Segment Number												
	I	II	III	IV	V	VI	VII	VIII	IX	X	XI	XII	XIII
Number of elements	1 + ae	3 + ae	1 + ae	1	1 + ae	1	1 + ae	1 + sp	2 + ae	1	1	1 + ae + sp	1
	XIV	XV	XVI	XVII	XVIII	XIX	XX	XXI	XXII	XXIII	XXIV	XXV	
Number of elements	1 + ae	1	1 + ae	1	1	1	1	1	2	2	2	4 + ae	

**Table 5:** Armature formula of the right male antennule of *Mongolodiaptomus longiserratus* sp. nov. The number of setae (Arabic numerals), aesthetascs (ae), spines (sp), and spiniform process (spr) is given. The Roman numerals refer to segment numbers.

	Segment Number										
	I	II	III	IV	V	VI	VII	VIII	IX	X	XI
Number of elements	1 + ae	3 + ae	1 + ae	1	1 + ae	1	1 + ae	1 + sp	2 + ae	1 + sp	1 + sp
	XII	XIII	XIV	XV	XVI	XVII	XVIII	XIX	XX	XXI	XXII
Number of elements	2 + ae	1 + spr	2 + ae + spr	2 + ae + sp	2 + ae + sp	2 + spr	1 + spr	2	2 + spr	2	4 + ae

**Table 6:** Armature formula of the swimming legs of *Mongolodiaptomus longiserratus* sp. nov. The quantity of setae (indicated by Arabic numerals) and spines (Roman numerals) is provided in the following sequence: outer-inner margin or outer-apical-inner margin.

	Coxa	Basis	Exp			Enp		
			1	2	3	1	2	3
P1	0-1	0-0	I-1	0-1	I-3-2	0-1	I-2-3	- - -
P2	0-1	0-0	I-1	I-1	I-3-3	0-1	0-2	2-2-3
P3	0-1	0-0	I-1	I-1	I-3-3	0-1	0-2	2-2-3
P4	0-1	1-0	I-1	I-1	I-3-3	0-1	0-2	2-2-3

**Table 7:** Morphological comparison of male characters from *Mongolodiaptomus parabirulai* sp. nov., *M. longiserratus* sp. nov., *M. birulai*, *M.* cf. *birulai*, and *M. formosanus*.

Male Characters	*M. parabirulai* sp. nov.	*M. birulai*	*M.* cf. *birulai*	*M. formosanus*	*M. longiserratus* sp. nov.
Collection localities and references	Yunnan, China This study	Harbin, China Rylov [19]	Philippines Lai et al. [24]	Taiwan Kiefer [15]	Cambodia and Vietnam This study
Right antennule: spine on segment 16	Short	No data	Short	Very short	Short
Right antennule: spiniform process on segment 20	Sickle-shaped, ~2/3 length of segment 21, with smooth outer margin	Sickle-shaped, ~1/2 length of segment 21, with smooth outer margin	Sickle-shaped, ~3/4 length of segment 21, with smooth outer margin	Knife-shaped, ~1/2 length of segment 21, with smooth outer margin	Sickle-shaped, longer than segment 21, with serrate outer margin
Ventral outgrowth(s) on right caudal ramus	Middle outgrowth large, plus two tiny knobs distally	Middle outgrowth extra-large	Middle outgrowth large or extra-large	Middle outgrowth small	Middle outgrowth small, plus two small knobs distally
Right P5 basis: shape and length: width ratio	Strongly robust trapezoid, ~1.1 × as long as wide	Sturdy trapezoid, ~1.5 × as long as wide	Strongly robust trapezoid, ~1.2 × as long as wide	Sturdy trapezoid, ~1.5 × as long as wide	Slender rectangle, ~1.7 × as long as wide
Right P5 basis: inner margin	With one longitudinal hyaline lamella	With one longitudinal hyaline lamella	With one longitudinal hyaline lamella	With one longitudinal hyaline lamella	With two (longitudinal and semicircular) hyaline lamellae
Right P5 Enp: shape and length	Sturdy triangular, ~1/3 length of Exp-2	Sturdy triangular, ~1/3 length of Exp-2	Sturdy triangular, ~1/3 length of Exp-2	Sturdy triangular, ~1/3 length of Exp-2	Slender truncated cone, ~2/3 length of Exp-2
Right P5 Exp-1: outer distal margin	Unproduced	Unproduced	Unproduced	Produced into acute tip	Produced into acute tip
Right P5 Exp-2: shape	With bulging inner margin, outer portion produced into wing-shaped expansion distally	No bulging inner margin, distal outer portion unproduced	With bulging inner margin, outer portion produced into wing-shaped expansion distally	With bulging inner margin, distal outer portion unproduced	No bulging inner margin, distal outer portion unproduced
Right P5 Exp-2: principal lateral spine	short, ~1/2 length of segment	short, ~1/2 length of segment	short, ~1/2 length of segment	short, ~1/2 length of segment	long, ~2/3 length of segment
Left P5 basis: inner margin	With a small spine	With a small spine	With a small spine	Without any spine	With a small spine
Left P5 Enp	two-segmented	one-segmented	one-segmented	one-segmented	two-segmented

**Table 8:** Morphological comparison of female characters from *Mongolodiaptomus parabirulai* sp. nov., *M. longiserratus* sp. nov., *M. birulai*, *M.* cf. *birulai*, and *M. formosanus*. Data on collection localities and references are the same as those in Table 7.

Female Characters	*M. parabirulai* sp. nov.	*M. birulai*	*M.* cf. *birulai*	*M. formosanus*	*M. longiserratus* sp. nov.
Antennules	Reaching far beyond end of caudal setae	Reaching ~end of caudal setae or slightly longer	Reaching ~end of caudal setae	Reach far beyond end of caudal setae	Reaching not beyond end of caudal setae
Overlap of thoracic and urosomal lobes	Yes	No	Yes	Yes	Yes
Genital double-somite	Strongly asymmetrical	Strongly asymmetrical	Strongly asymmetrical	Strongly asymmetrical	Almost symmetrical
Genital double-somite: proximal portion	Left portion largely dilated	Left portion largely dilated	Left portion largely dilated	Left portion largely dilated	Left and right portions equally dilated
Genital double-somite: left and right spines	Left spine larger than right spine; left spine pointed laterally	Left spine larger than right spine; left spine pointed laterally	Left spine larger than right spine; left spine pointed laterally	Left spine larger than right spine; left spine pointed laterally	Both spines equal in size; left spine pointed distally
P5	Symmetrical	Symmetrical	Nearly symmetrical	Asymmetrical	Asymmetrical
P5 basis: seta on outer margin	Long, reaching ~3/4 of Exp-1	Very short, reaching ~1/10 of Exp-1	Very short, reaching ~1/10 of Exp-1	Short, reaching ~1/2 of Exp-1	Short reaching ~1/3 of Exp-1
P5 Enp	two-segmented, reaching nearly the same length of Exp-1	one-segmented, reaching ~ 2/3 of Exp-1	two-segmented, reaching ~2/3 of Exp-1	one-segmented, reaching ~2/3 of Exp-1	two-segmented, reaching nearly the same length of Exp-1

## Data Availability

The data presented in this study are available in the article.
